# The Rothman Index Does Not Predict a Successful Extubation in the Neurosurgical Critical Care Unit

**DOI:** 10.7759/cureus.16339

**Published:** 2021-07-12

**Authors:** Abdullah Ghali, Mouhamed Nashawi, Justin Johal, Josh Learned, Mohammed T Al-Hamaydeh, Ali Seifi, Shaheryar Hafeez

**Affiliations:** 1 Neurosurgery, University of Texas Health Science Center at San Antonio, San Antonio, USA; 2 Internal Medicine, University of Texas Health Science Center at San Antonio, San Antonio, USA; 3 Anesthesia and Critical Care, University of Washington, Seattle, USA

**Keywords:** reintubation, rothman index, extubation, intubation, neuro-critical care

## Abstract

Background

Identification of risk factors associated with successful extubation in neurosurgical critical care units (NSICUs) has been elusive due to the complex nature of neurocritical care injuries and patient factors. Traditional risk factors for extubation were shown to have poor predictive value in neurocritical care patients as compared to mixed ICU patients. The aim of this study was to determine if any risk factors, including the Rothman Index, could reliably predict successful extubation in a large sample size of neurocritical care patients.

Methods

We retrospectively analyzed 610 consecutively intubated patients in an NSICU while collecting variables of interest in airway management. Furthermore, Rothman Indices were collected immediately after intubations and extubations. A paired t-test of the immediate changes in Rothman Indices after airway manipulation was compared in patients who needed reintubation. In a smaller cohort of 88 patients, in whom complete data points existed for airway management, we performed a principal component analysis (PCA) to determine which risk factors were associated with extubation success when indexed with the magnitude of the Rothman Index.

Results

In 610 consecutively intubated patients, the mean pre-intubation Rothman Index average was 41.0 compared to the mean post-extubation Rothman Index was 35.4 (p<0.0001). Compared to those who were re-intubated, the Rothman Index did not correlate well with the prediction of extubation (p=0.355). Furthermore, an analysis of the PCA plot showed that a higher respiratory rate, longer length of stay, shorter length of intubation, and smaller cuff leak percentage were identified as risk factors associated with reintubation. Age and change in rapid shallow breathing index (RSBI) did not correlate with reintubation.

Conclusion

The Rothman Index does not predict extubation success in patients in an NSICU. Risk factors associated with reintubation were respiratory rate, length of stay, length of intubation, and cuff leak percentage. Reintubation rates in our single-center NSICU are on par with general critical care populations.

## Introduction

In the critical care setting, mechanical ventilation via ventilator is a mainstay intervention that saves lives in patients who experience difficulty breathing. Particular indications may warrant auxiliary support to promote adequate ventilator function. Notably, interventional support via tracheal intubation is commonly performed on critically ill patients in need of breathing support with a ventilator, as intubation promotes the airway patency needed for gas flux while mitigating asphyxiation or airway obstruction. In complicated airway access scenarios, tracheostomy makes mechanical ventilation amenable by offering a direct approach to the trachea. It should be noted that mechanical ventilation is not devoid of risks. These include ventilator-associated pneumonia (VAP), pulmonary barotrauma, vocal cord injury, tracheal stenosis, oxygen toxicity, and reductions in cardiac output secondary to altered hemodynamics, to name a few [1-2]. Usually, if a prognosis is deemed to trend towards positive recovery relative to initial admission status, the goal for mechanically ventilated patients who are intubated is to provide a period of respiratory support until prospective extubation and ventilator liberation is viable. This management approach allows a patient to retain the ability to facilitate vital gas exchange processes necessary for life and appropriates time for potential clinical restoration to a status of more independent breathing while recovering. While this respiratory arrangement is essential in these scenarios for survival in the critically ill, reducing the time interval between initial mechanical ventilation to extubation is vital in the reduction of healthcare costs and the improvement of patient outcomes by decreasing complications such as VAP. Despite medical optimization, it is estimated that 5%-20% of patients liberated from mechanical ventilation in intensive care units (ICUs) require reintubation [3-5]. This predicament consumes institutional resources, prolongs the window for complications in patients, and delays discharges or downgrades to facilities that may cater to a recovering patient more adequately.

Therefore, there is a vested interest in quality improvement and healthcare delivery to investigate circumstances related to reintubation. In addition to an expanding body of knowledge in critical care pathologies, technological innovations, such as surveillance metrics, are available at some institutions to aid in guiding providers to determine successful extubation opportunities, and, by extension, opportunities in which one would be reticent to extubate given failed extubations and reintubations. Moreover, the critically ill patient populous is diverse in their demographics, comorbidities, and floor location, which means that the circumstances in extubation and intubation decision-making vary accordingly. One such setting is the neurosurgical intensive care unit (NSICU). Reintubation rates, tracheostomy rates, and factors influencing the exigency for reintubation in the NSICU have not been extensively studied, and reports of such are scant in the literature. Such deficiencies in investigative sources limit the propensity for providers in these settings to optimize successful extubations. Moreover, the surveillance metrics mentioned previously that have been purported to augment the optimization of extubation and ventilator liberation do not correlate well with extubation success when applied to patients in the NSICU setting [5-7]. As iterated previously, these may due to unique factors of this patient population influencing their liberation from mechanical ventilation in the NSICU. These include, but are not limited to, inability to follow commands, neuromuscular weakness, autonomic dysfunction, and cranial nerve dysfunction [7-9]. The surveillance metrics used as a supplementary aide (with clinical judgment) regarding the extubation decision-making process have found increasing prominence in their use via their construct as computer algorithms embedded as tools within the graphical user interface (GUI) of electronic medical record (EMR) software programs. They can be shown as alert messages, text headers, or accessible patient information, akin to a tab of a patient's past medical history, with the ability of dynamic change to assist in clinical care decision-making in real-time, as information is processed routinely within the EMR software. The Rothman Index (RI) is one of the artificial intelligence (AI)-based algorithms. It is a data metric that compiles multiple data points within a patient’s electronic health record in real-time and computes a real number whose magnitude ranges from -91 to 100, with positive numbers of larger magnitude implying a better health status within that time point [10-12]. It relies on various vital sign data points, heart rhythms, nursing reports, and the magnitude of quantitative laboratory specimens (e.g., sodium, serum creatinine, etc.). Acute declines in the magnitude of RI imply acute decompensation. In theory, we may notice an RI decline in an extubated patient who is in immediate respiratory distress if the RI projects prognosis as designed. The dataset or algorithm it bases its projections on has not been fully elucidated, and its validity in the NSICU has not been extensively validated, raising inquiries on its utilization in the NSICU. However, this metric was chosen, as it was part of our institution's electronic medical record, and this study sought to determine if it could be used to predict extubation success in patients in the NSICU. To offer parallel validity, we collected data associated with prognosis and airway dynamics to compare as a template relative to the RI for cross-validation. Thus, the goals of this retrospective case study are to study the factors that influence reintubation in the NSICU and whether the Rothman Index will accurately predict the clinical status of patients in the NSICU by assessing its validity directly to clinical status.

## Materials and methods

In this retrospective study, several collected factors associated with mechanical ventilation and airway physiology were collected on 610 consecutively intubated patients admitted to the NSICU from August 2017 to June 2019 in a large, tertiary care center in South Texas. Among the data collected for each patient were: demographic factors (age and sex), clinical factors (BMI, primary brain injury type, subsequent injuries acquired during ICU stay, duration of intubation, duration of reintubation, etiology of reintubation, total length of stay, ΔRothman in the events immediately proceeding extubation with subsequent reintubation, and variables assessing pulmonary status such as spontaneous breathing trial (SBT), rapid shallow breathing index (RSBi), respiratory rate (RR), tidal volume (VT), forced vital capacity (FVC), and cuff leak percentage. A paired t-test was conducted to determine if the Rothman Index, by nature of its definition, could accurately assess if an extubation warranted a reintubation based on the magnitude of the variable and if a reintubation was successful based on the Rothman Index change. The proportion of reintubations and tracheostomies within this unique cohort were identified and reported. Moreover, a smaller cohort of 88 extubated patients who had complete pulmonary records regarding the previously variables separately underwent principal component analysis (PCA) to elucidate variables and associations of interest in reintubation with relative comparison to the Rothman Index.

## Results

In 610 consecutively intubated patients, the mean pre-intubation Rothman Index was 41.0 while the mean post-extubation Rothman Index was 35.4 (p<0.0001). The mean difference in the Rothman Index at intubation and during extubation among all patients who were mechanically ventilated was -5.6 (P <0.05). Amongst the reintubated cohort, the mean difference in the Rothman Index was -1.2, (P=0.355). In the total cohort of 610 intubated patients, 38 (6.2%) of patients required reintubation. Sixty-one (10%) of patients required tracheostomy. Of the 38 patients who underwent reintubation, 11 underwent tracheostomy (1.8% of the total cohort and 28.9% of the reintubated cohort). See Table 1 for the variable means of patients admitted to the NSICU.

**Table 1:** Variable Means of Patients Admitted to NSICU BMI: body mass index; FVC: forced vital capacity; RSBi: rapid shallow breathing index; NSICU: neurosurgical intensive care unit

	BMI	Cuff Leak %	FVC (L)	Age (Years)	Vt (L)	Length of Stay (days)	Length of Intubation (days)	RR	∆Rothman	∆RSBi
610 Patient (Total)	28.4	49.7	1.2	55.7	0.492	6.8	4.5	19.0	-5.8	-0.17
88 Patients (Comprehensive Variables)	29.33	48.5	1.2	54.3	0.482	8.1	4.0	19.1	-6.6	2.11
38 Reintubated Patients	27.0	44.9	1.0	56.1	0.469	13.5	4.0	19.3	-2.2	-0.78

**Figure 1:**
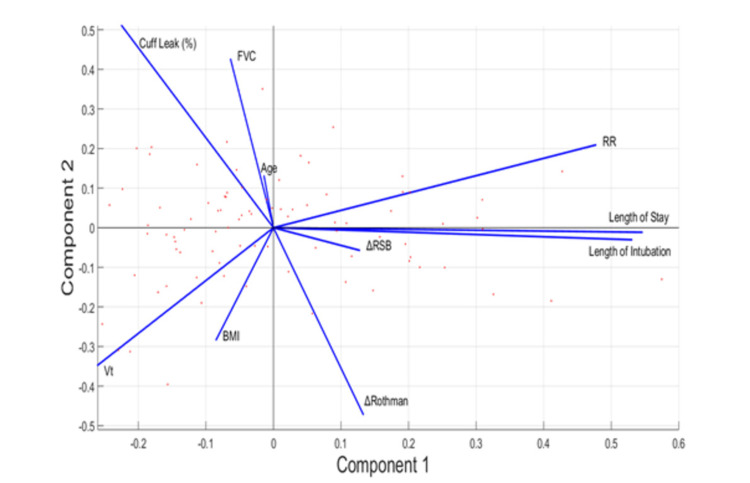
Principal Component Analysis Biplot of 88 Cohort Patients

The secondary portion of this study aimed to examine the reasons for extubation success with a relative comparison to the interpretation of the Rothman Index for the validity test. In this study, 88 subjects had complete data variables amenable for indexed analysis. The 10 variables collected had associations with extubation success and serve as overall indicators of airway physiology. In principal component analysis, the number of principal components (a working definition that is a partition of the analyzed dataset) correlates with the variability of the dataset and how many dimensions that the data can be visualized in. For example, retaining all principal components will keep 100% of the variability of the dataset. It is of interest in some datasets to retain a critical amount of variability within a small number of principal components, such as two or three, to be able to visualize the dataset in a two-dimensional or three-dimensional plot, which is more amenable to visualization. In this dataset, it was shown that 94.8% of variability could be retained in the first two principal components, with 96.6% variability expressed in the first three components, owing credence to the visualization of these patients with robust affinity in a two-dimensional biplot for visualization, which can be seen below (Figure 1). In this visualization process, factors were identified that were strongly associated with reintubation status in this cohort to be respiratory rate, length of stay, length of intubation, and cuff leak percentage as is evident by the magnitude of the vectors in the PCA plot. Interestingly, factors with the poor association of the reintubation behavior expressed among this cohort include age and the change in RSBi. 

## Discussion

This work represents one of the reports of using a computer algorithm, the Rothman Index, which does not reliably predict improving or worsening patient conditions to guide the likelihood of extubation in NSICU. It is important to note, however, that the tool itself is used as an adjunct to clinical decision-making, rather than a replacement. Lower Rothman Indices have been correlated with poorer outcomes in multiple settings [10-13]. However, appropriate extubation is considered to be a process correlated with improved health outcomes, as it represents a patient has reached improved cardiopulmonary and neurological functionality. In fact, this result showed the opposite result, and the Rothman Index would have predicted extubation led to a poorer clinical state. This raises a serious inquiry as to whether this index should be used for this particular patient population. It may not represent an appropriate surveillance metric in the NSICU given the dataset it was trained on or the algorithmic computations it uses to assess a metric and whether or not these calculations are appropriate for the unique nature of NSICU patients. This study also highlights the importance of a clinician’s skills at a patient’s bedside as opposed to relying on an algorithm that generates a variable to correlate with clinical conditions. Caution should be taken to substitute clinical judgment and show dependence on a variable that may not necessarily be accurate. Clinical skills and training remain in the best interest of patients. We recommend that these metric tools be cautiously used as a support for clinical decision-making but never as core evidence to guide decision-making.

Interestingly, Figure 1 has also elucidated interesting correlations in a cohort of intubated patients. In analyzing a bi-plot, a rule of thumb is that correlation is generally associated with a smaller angle between two vectors on the biplot, such as length of stay and length of intubation, which makes intuitive sense. Once the angle between two vectors approaches 180 degrees, there is an inverse correlation such as seen in respiratory rate and tidal volume for fixed pulmonary ventilation (PV = VT X RR). Orthogonal vectors generally indicate a weak association between variables, as is seen between the change in the Rothman Index and the change in respiratory rate, the latter an obvious predictor of mortality and health outcomes. These associations raise more questions into the factors that are associated with reintubation in the NSICU: why is a change in Rothman inversely correlated with the cuff leak percentage, i.e., the sicker a patient is, the lower the cuff leak; and what is the role surveillance metrics, such as the Rothman Index, have on implementing interventions in patients who are acutely ill. Moreover, the clustering of patients represents similar aggregate trends in the hospitalization course, whereas geometric outliers represent interesting cases that may give us insight into how trends in the NSICU differ amongst patients (the red dots that appear far away from the center). 

Additionally, this series presents the largest examination of reintubations in a neurosurgical critical care setting and establishes a reintubation rate of 6.2%. This is comparable to previously reported institutions with different clinical settings such as the trauma ICU (27/405, or 7%) at an academic hospital [14]. However, other sources report reintubation rates as high as up to over 50% in some medical ICUs [15]. The difference in these rates, when compared with our institution, may be explained by prolonged intubation and aggressive monitoring - where extubation is warranted only when indicated per institutional protocol. Another limitation of this study is that we did not control for patient diagnoses within the cohort. We encourage further research and investigation in regard to the efficacy of the Rothman Index for specific diagnoses such as subarachnoid hemorrhage.

## Conclusions

Patients with conditions that warrant intubation in the neurocritical care setting have their own, unique, specific considerations that must be taken into account when making the decision to extubate to minimize reintubation and allow for successful mechanical ventilation liberation. We have demonstrated that the factors associated with reintubation are respiratory rate, length of stay, length of intubation, and cuff leak percentage, which are congruent, complicated airways. Nevertheless, the Rothman Index, when compared to these variables, in addition to the expected change in magnitude for the Rothman index within a specific airway manipulation (e.g., change in magnitude upon intubation or extubation) are not congruent. Therefore, we believe the Rothman Index, as currently constructed, cannot be used to determine whether an extubation will be successful in the NSICU. This warrants prudence regarding this surveillance metric in this setting and may necessitate an overall investigation in surveillance metrics within different clinical settings for optimized decision-making.
